# Setting time of construction gypsum, dental plaster, and white orthodontic gypsum

**DOI:** 10.34172/joddd.2020.036

**Published:** 2020-09-21

**Authors:** Imelda Darmawan, Octarina Willy, Johan Arief Budiman

**Affiliations:** ^1^Department and Institution, Faculty of Dentistry, Trisakti University, Jakarta, Indonesia

**Keywords:** Construction gypsum, Dental plaster, Final setting time, Initial setting time, White Orthodontic gypsum

## Abstract

**Background.** Dental plaster, white orthodontic gypsum, and construction gypsum have β-hemihydrate particles. Setting time is an essential property of dental gypsum, which can affect the strength of the material. This research aimed to compare construction gypsum, dental plaster, and white orthodontic gypsum’s initial and final setting times.

**Methods.** Three groups were included in this experimental laboratory study: construction gypsum (A), dental plaster (B), and white orthodontic gypsum (C). Each group consisted of 10 samples. Gypsum manipulation consisted of using 120 gr of powder and 60 mL of water. Gypsum powder and water were mixed using a gypsum mixer at 120 rpm. A homogeneous mixture was poured into a mold, and the setting time was measured using a Gillmore needle, according to ASTM C266-03. The initial setting time test was measured using 113.4 grams and a 2.12-mm needle. The final setting time was measured using 453.6 grams and a 1.06-mm needle. This test was repeated until the needle failed to penetrate the gypsum’s surface. All the data were analyzed with one-way ANOVA and post hoc Tukey tests using SPSS 23.

**Results.** The average initial setting time for groups A, B, and C were 10.39±1.19, 16.17±1.40, and 24.46±1.51, respectively. The average final setting time for groups A, B, and C were 15.97±0.79, 24.31±0.88) and 33.37±0.66, respectively. One-way ANOVA and post hoc Tukey tests showed significant differences in the initial and final setting times between the three groups (P<0.05).

**Conclusion.** There were differences in setting time between dental plaster, white orthodontic gypsum, and construction gypsum. The construction gypsum’s setting time is suitable as a type II dental gypsum, according to ADA No.25.

## Introduction

Gypsum is one of the natural minerals that contain calcium, hydrogen, water, and sulfur, known as calcium sulfate dihydrate (CaSO_4_.2H_2_O).^1^ Gypsum products are available in the form of a fine white powder which has undergone calcination or heating at a temperature of 110‒130°C in the open air. This process causes some of the gypsum material to become dehydrated to calcium sulfate hemihydrate (CaSO_4_.2H_2_O) called Plaster of Paris in the form of β-hemihydrate.^2,3,4^

Construction gypsum is used in the manufacture of gypsum boards or ceilings in the building interior industry.^5^ Gypsum is the material of choice because it has a low price, is easy to install, and has characteristics that meet the criteria as building materials. Construction gypsum material is in the form of β-hemihydrate with the characteristics of low density and high porosity.^5^ This gypsum form of hemihydrate becomes hydrated when mixed with water and undergoes the process of setting. At the same time, there is an increase in the strength of the material because the final microstructure of set gypsum material can affect the gypsum stiffness.^6,7^

Type II dental gypsum or dental plaster contains β-hemihydrate particles and is used as a study model material, a set-up material for the working model on articulators, and dental laboratory material. Orthodontic white gypsum containing β-hemihydrate particles is used as a study model in orthodontics to provide a three-dimensional picture of the patient’s occlusion, making it easier for dentists to determine treatment plans.^8^ The material in dentistry should have the characteristics that affect the strength, such as setting time.^9^ The setting process starts when the gypsum powder is mixed with water (hydration). There are two time intervals: initial setting time and final setting time, when setting time is in progress. The time from mixing gypsum powder with water until half-hardened gypsum consistency is achieved is called the initial setting time, while the final setting time is the time from mixing until the material hardens and can be removed from the mold. Humidity can influence the setting process. The setting time can be measured by penetration testing using the Gillmore needle.^10,11^

Based on the Ministry of Health, Republic of Indonesia Regulation Article No. 1189/MoH/REG/VIII/2010 concerning the production of medical devices and household health supplies and Ministry of Health, Republic of Indonesia Regulation Article No. 1190/MoH/REG/VIII/2010 concerning distribution permits for medical devices and household health supplies,^12,13^ all the dental materials circulating in Indonesia must have a distribution permit, including imported dental plaster and white orthodontics gypsum. These regulations make it difficult for both imported gypsums to be found on the Indonesian market because it is quite difficult to obtain the permit to distribute medical devices. The attempt to produce local gypsum products, with wet calcination method using autoclave, had been compared.^14^ The research raises the thought of using construction gypsum, abundant in Indonesia, as an alternative material for dental plaster and white orthodontics gypsum. Construction gypsum has the same basic material and molecular shape as dental plaster and white orthodontics plaster. This study aimed to compare construction gypsum, dental plaster, and white orthodontic gypsum’s initial and final setting times.

## Methods

This laboratory study was conducted at the Dental Materials and Testing Centre of Research (DMT-Core) at our institution in October-November 2019. The samples used were divided into three groups: construction gypsum, dental plaster, and white orthodontic gypsum. The 10 gypsum block samples for each group were set based on the Federer formula.^7^ The tools used in this research were Gillmore needle setting time test equipment, molds, measuring cups, automatic mixers, vibrators, digital scales, and water temperature thermometers. The materials used in this study were Aquadest, APLUS^®^ construction gypsum, Pro-BASE^®^ dental plaster, and SIRIUS^®^ white orthodontic gypsum.

Manipulating the gypsum block sample to be tested began by weighing 120 grams of gypsum powder and taking 60 mL of water. Water was firstly put in a bowl; then, the gypsum powder was slowly added and let stand for 30 seconds. When gypsum powder contacted water, the stopwatch calculation started. Mixing was carried out for 60 seconds using an automatic mixer to obtain a homogeneous mixture.^2^ The next stage consisted of pouring the mixture into a block-mold, which was then vibrated with a vibrator so that the surface of the gypsum sample was flat. The gypsum block sample that was poured into the mold was tested by setting the time under the Gillmore needle to test the initial setting time.

The Gilmore needle was positioned vertically against the sample with the needle tip in contact with the sample’s surface. The needle was then released until it penetrated the sample. The time of the needle for penetrating the sample was 15 seconds. After 15 seconds, the needle was removed; then, the gypsum attached to the needle tip was cleaned with tissue paper and positioned for the next penetration area. The penetration of the needle was carried out around the sample to get a different puncture area. The needle was removed in 15 seconds; therefore, the one-time penetration took 30 seconds. This continued until the needle could not leave a trace on the surface of the gypsum block sample. When the needle to test the initial setting time could not leave a trace on the gypsum surface, the test proceeded with positioning the sample under the Gillmore needle to test the final setting time. The process to test the final setting time was the same as the initial setting time.^2^

Statistical analysis of the data in this study consisted of one-way ANOVA (SPSS 23). If the results of the data analysis produced significant differences, then a further test was conducted, i.e., post hoc Tukey test.

## Results

Table 1 presents the test results of construction gypsum and type II dental gypsum’s setting time. The lowest mean of initial setting time was found in the construction gypsum with 10 minutes and 39 seconds; the highest mean was found in the SIRIUS^®^ dental gypsum with 24 minutes and 46 seconds. The lowest final setting time was also found in the construction gypsum with 15 minutes and 97 seconds; the highest mean value in the dentistry gypsum SIRIUS^®^ was 33 minutes and 37 seconds.

**Table 1:** Comparison of average results of measurement of construction gypsum setting time and dental gypsum

**Gypsum**	**N**	**Initial setting time**	**Final setting time**
		**± SD (min)**	**± SD (min)**
**APLUS** ^®^	10	10.39±1.19	15.97±0.79
**Pro BASE** ^®^	10	16.17±1.40	24.31±0.88
**SIRIUS** ^®^	10	24.46±1.51	33.37±0.66

This study used one-way ANOVA after it was ensured that the data were distributed normally. The initial and final setting time measurement data in this study fulfilled all the requirements to perform one-way ANOVA. ANOVA showed the significance of the initial setting time variable, and the final setting time showed a significant initial setting time difference between the construction gypsum and type II dental gypsum (dental plaster and white orthodontic) (P<0.05). Further analyses were carried out with post hoc Tukey tests at a significance level of P<0.05. Tables 2 and 3 show significant differences in initial setting time and final setting time in all the tested groups (P<0.05). Therefore, it can be concluded that all the groups exhibited significant differences.

**Table 2:** Statistical analysis of initial setting time of tested gypsum groups with one-way ANOVA and post hoc Tukey tests

**Gypsum**	**APLUS** ^®^	**Pro BASE** ^®^	**SIRIUS** ^®^
**APLUS** ^®^	-	0.000*	0.000*
**Pro-BASE** ^®^	-	-	0.000*
**SIRIUS** ^®^	-	-	-

*P<0.05 significance

**Table 3:** Statistical analysis of final setting time of tested gypsum groups with one-way ANOVA and post hoc Tukey tests

**Gypsum**	**APLUS** ^®^	**Pro BASE** ^®^	**SIRIUS** ^®^
**APLUS** ^®^	-	0.000*	0.000*
**Pro-BASE** ^®^	-	-	0.000*
**SIRIUS** ^®^	-	-	-

*P<0.05 significance

## Discussion

Table 1 presents the average setting times of construction gypsum (APLUS^®^) and type II dental gypsum (Pro-BASE^®^ and SIRIUS^®^). APLUS^®^ gypsum has an average initial setting time of 10 minutes and 39 seconds, with a final setting time of 15 minutes and 97 seconds. This shows that the construction gypsum (APLUS^®^) setting time meets ADA #25 standard specifications (8‒16 minutes). Pro-BASE^®^ gypsum has an average initial setting time of 16 minutes and 17 seconds, with a final setting time of 24 minutes and 31 seconds. These results indicate that the Pro-BASE^®^ gypsum setting time does not meet the ADA #25 standard. SIRIUS^®^ gypsum has an average initial setting time of 24 minutes and 46 seconds and a final setting time of 33 minutes 37 seconds. The average setting time of SIRIUS^®^ gypsum does not meet ADA #25 standard. Other research on local gypsum products showed that the average setting time of the self-made gypsums was around 8 minutes and 7 seconds and 3 minutes and 40 seconds.^14^ The study above also reported an average setting time of 20 minutes and 21 seconds for dental plaster and 10 minutes and 34 seconds for dental stone.^14^

Table 1 shows that the average initial setting time value was lowest in APLUS^®^ construction gypsum and highest in SIRIUS^®^ gypsum. The final time setting values of three type gypsums in this study were different, as shown in Table 1. This time setting difference can be influenced by the amount of crystallization core in the gypsum. During the setting reaction, a nucleation process occurs between the calcium (Ca^2+^) and sulfate (SO_4_-) ions, which form a molecular bond. When these two molecules come together, a nucleus of crystallization will emerge. The higher the number of crystallization nuclei, the faster the formation of dihydrated crystals so that gypsum will harden faster.^10^ The factor making the average setting time of type II dental gypsum longer in this study than the ADA #25 standard (8‒16 minutes) is the hygroscopic nature of gypsum material (drawing water from the air). Gypsum storage contaminated with air can attract water and cause low solubility of dihydrate molecules, increasing the setting time of gypsum. Based on ISO 6873, the standard for Dental gypsum storage is 50% ± 10%.^15^ Other research on gypsum material found that Indonesia’s humidity level is quite high, reaching 70%.^16^ The annual weather report (2019) showed that in Jakarta (the capital of Indonesia) January is on average the most humid; September is the least humid month; and the average annual humidity percentage is 80.0%.^17^ High humidity can affect the properties and reduce the quality of gypsum material.^16^ Gypsum with hygroscopic properties will become moist (damp) in places with high humidity.^18^ The water content in gypsum powder reduces the gypsum hemihydrate molecule, increasing the setting time of the material.^11^

SIRIUS^®^ gypsum’s setting time was the highest compared to the other two gypsum products. A factor that increases the setting time of SIRIUS^®^ gypsum is the powder-to-water ratio when gypsum is manipulated. This study used a powder-to-water ratio of 2:1 following the Type II dental gypsum ratio. SIRIUS^®^ gypsum manufacturer recommends a 3:1 powder-to-water ratio, which is a type III gypsum ratio.^10^ SIRIUS® gypsum is thought to be an Orthodontic plaster containing type II gypsum and type III gypsum.^19^ Α-hemihydrate particles are low in porosity so that they do not require as much gypsum as water compared to β-hemihydrate particles.^20^ The excess water used when mixing would make the gypsum’s setting time longer.^18^ This gypsum material contains α-hemihydrate particles with a denser and less porous particle structure so that it can be used for manufacturing study models because it re-produces accurate oral anatomy.^21^ The long setting time for SIRIUS^®^ gypsum is because the orthodontic gypsum’s working time is longer than other gypsums, aiming to achieve more accurate study models.^10^

Another factor influencing the difference in gypsum setting time in this study is composition. The three tested gypsums have different manufacturers, with different percentages of calcium sulfate hemihydrate and other chemicals in their structure. Several chemicals are used by manufacturers to manipulate the setting time for a gypsum product. A material often used to prolong the setting time is 1‒2% borax. Borax can form a coating on a hemihydrate molecule so that it cannot contact water, decreasing the solubility of the hemihydrate (i.e., increasing the setting time). The material often used to speed up setting time is 2‒3% potassium sulfate. These chemicals can make hemihydrate molecules more soluble when mixed with water.^2,10,22^

This study showed the lowest initial setting time and final setting time in APLUS^®^ construction gypsum, while the highest initial setting time and final setting time were recorded in SIRIUS^®^ type II dental gypsum. The difference in values in each gypsum group can be influenced by the amount of crystallization core, ambient humidity, and composition.^2^

## Conclusion

Construction gypsum and type II dental gypsum (dental plaster and White orthodontic) have different initial and final setting times. The APLUS^®^ gypsum’s setting time meets the ANSI-ADA standard #25. Construction gypsum (APLUS^®^) can be used as a substitute for type II dental gypsum. Further studies should be conducted to compare the other properties of these three gypsum materials.

## Authors’ Contributions

ID was responsible for reviewing the literature and performing the experiments as fulfillment of requirements for her degree. OW was responsible for the experiment design and hypothesis and contributed to the discussion. JAB conceived the idea and contributed to prepare and wrote the manuscript. All the authors have read and agreed to the published version of the manuscript.

## Acknowledgments

Thanks to DMT-Core Faculty of Dentistry, Trisakti University for permitting this research to be carried out and to Rosalina Tjandrawinata, PhD, and Dewi Liliany, MSi, for the scientific input to make this research possible.

## Funding

Financial support was provided by the authors.

## Competing Interests

The authors declare no competing interests with regards to the authorship and/or publication of this article.

## Ethics approval

Not applicable.
